# A native IgE in complex with profilin provides insights into allergen recognition and cross-reactivity

**DOI:** 10.1038/s42003-022-03718-w

**Published:** 2022-07-27

**Authors:** Benjamín García-Ramírez, Israel Mares-Mejía, Annia Rodríguez-Hernández, Patricia Cano-Sánchez, Alfredo Torres-Larios, Enrique Ortega, Adela Rodríguez-Romero

**Affiliations:** 1 Instituto de Química, Universidad Nacional Autónoma de México, Circuito Exterior, Cd. Universitaria, Coyoacán, Ciudad de México, 04510 Mexico; 2 Instituto de Fisiología Celular, Universidad Nacional Autónoma de México, Circuito Exterior, Cd. Universitaria, Coyoacán, Ciudad de México, 04510 Mexico; 3 Instituto de Investigaciones Biomédicas, Universidad Nacional Autónoma de México, Circuito Exterior, Cd. Universitaria, Coyoacán, Ciudad de México, 04510 Mexico

**Keywords:** X-ray crystallography, Biophysical chemistry

## Abstract

Allergies have become a rising health problem, where plentiful substances can trigger IgE-mediated allergies in humans. While profilins are considered minor allergens, these ubiquitous proteins are primary molecules involved in cross-reactivity and pollen-food allergy syndrome. Here we report the first crystal structures of murine Fab/IgE, with its chains naturally paired, in complex with the allergen profilin from *Hevea brasiliensis* (Hev b 8). The crystallographic models revealed that the IgE’s six complementarity-determining regions (CDRs) interact with the allergen, comprising a rigid paratope-epitope surface of 926 Å^2^, which includes an extensive network of interactions. Interestingly, we also observed previously unreported flexibility at Fab/IgE’s elbow angle, which did not influence the shape of the paratope. The Fab/IgE exhibits a high affinity for Hev b 8, even when using 1 M NaCl in BLI experiments. Finally, based on the encouraging cross-reactivity assays using two mutants of the maize profilin (Zea m 12), this antibody could be a promising tool in IgE engineering for diagnosis and research applications.

## Abstract (web summary)

The crystal structures of the murine IgE antibody in complex with highly cross-reactive profilins are reported, and the data of cross-reactivity among allergen mutants with antibodies may prove helpful for research applications in allergy therapeutics.

## Introduction

Numerous substances can trigger IgE-mediated allergies in humans, acting as haptens or antigens^1–3^. Allergens are not substances produced by organisms to cause allergies; they are molecules with specific biological functions^4^. Different organisms carry out the same biological processes, and many of them are executed by proteins with highly conserved amino acid sequences and 3D structures. These similarities could produce cross-reactivity towards allergens from different sources in patients^4^, i.e., IgE can recognize a protein similar to the one that originated the sensitization, giving rise to an allergic reaction.

Profilins are excellent examples of allergen cross-reactivity. They are ubiquitous proteins expressed in every eukaryotic cell and some archaea^5^. They also share high sequence identity and thus are considered panallergens^6^. Profilins are involved in multiple cellular functions, such as motility, through regulation of the polymerization of actin microfilaments, binding proteins with proline-rich domains, and binding phosphatidylinositol, among others. Profilins are 12–17 kDa proteins, with 100–153 amino acids^7^ that vary depending on the organism of origin and the isoform^8^. In general, these proteins maintain a high 3D structure and sequence identity, which is approximately 70% among plant profilins^9^. Therefore, in patients suffering respiratory allergies, cross-reactivity between profilins from foods and aeroallergens could induce oral-allergy syndrome or even anaphylaxis.

On the other hand, IgE is the primary antibody associated with allergies. Although, to date, there are no published crystallographic structures of intact IgE, free or in complex with an allergen, negatively stained microscopy, and small-angle X-ray scattering (SAXS) studies have exhibited significant rigidity of the IgE molecule^10^. The dynamics, allosteric properties, and flexibility studies rely on theoretical models of IgE bound to its high-affinity receptor. While there is vast information on the interactions between IgE-Fc and the FcεRI and CD23 receptors^11–15^, there are just a few examples of how recombinant Fab/IgE recognizes allergens^16–18^ and only one report on the structure of a native Fab/IgE; however, this antibody binds a hapten^19^. Concerning these few examples, the crystal structures of the allergens Bos d 5 (beta-lactoglobulin)^16^ and Phl p 2^17^ in complex with recombinant Fabs/IgEs show planar surfaces of interaction (890 and 855 Å^2^, respectively), and both IgEs were obtained in mammalian expression systems.

Here, we report the first crystal structures of the complex between a murine Fab/IgE (heavy and light chains naturally paired) that recognizes the allergen profilin from natural rubber latex (Hev b 8)^20^. This profilin is involved in the latex-fruit-pollen syndrome and oral-allergy syndrome and is highly cross-reactive^21^. Glycosylated Fab/IgE 2F5 exhibits an affinity for rHev b 8 in the medium-nanomolar range. Furthermore, IgE 2F5 can bind the allergen even after two annealing cycles. The structures of the free Fab/IgE 2F5 and its complex with rHev b 8 in two different conformations revealed the residues involved in the paratope-epitope interaction and provided insight into how an IgE recognizes an allergen and the structural basis of cross-reactivity.

## Results

To analyze the paratope-epitope interactions of the Fab/IgE 2F5-rHev b 8 complex, we determined two crystal structures of the complex and one of the Fab/IgE free, and we performed different characterization experiments to confirm the allergenic determinants.

### The IgE 2F5 recognizes profilin rHev b 8 after two annealing procedures

**Fig. 1:**
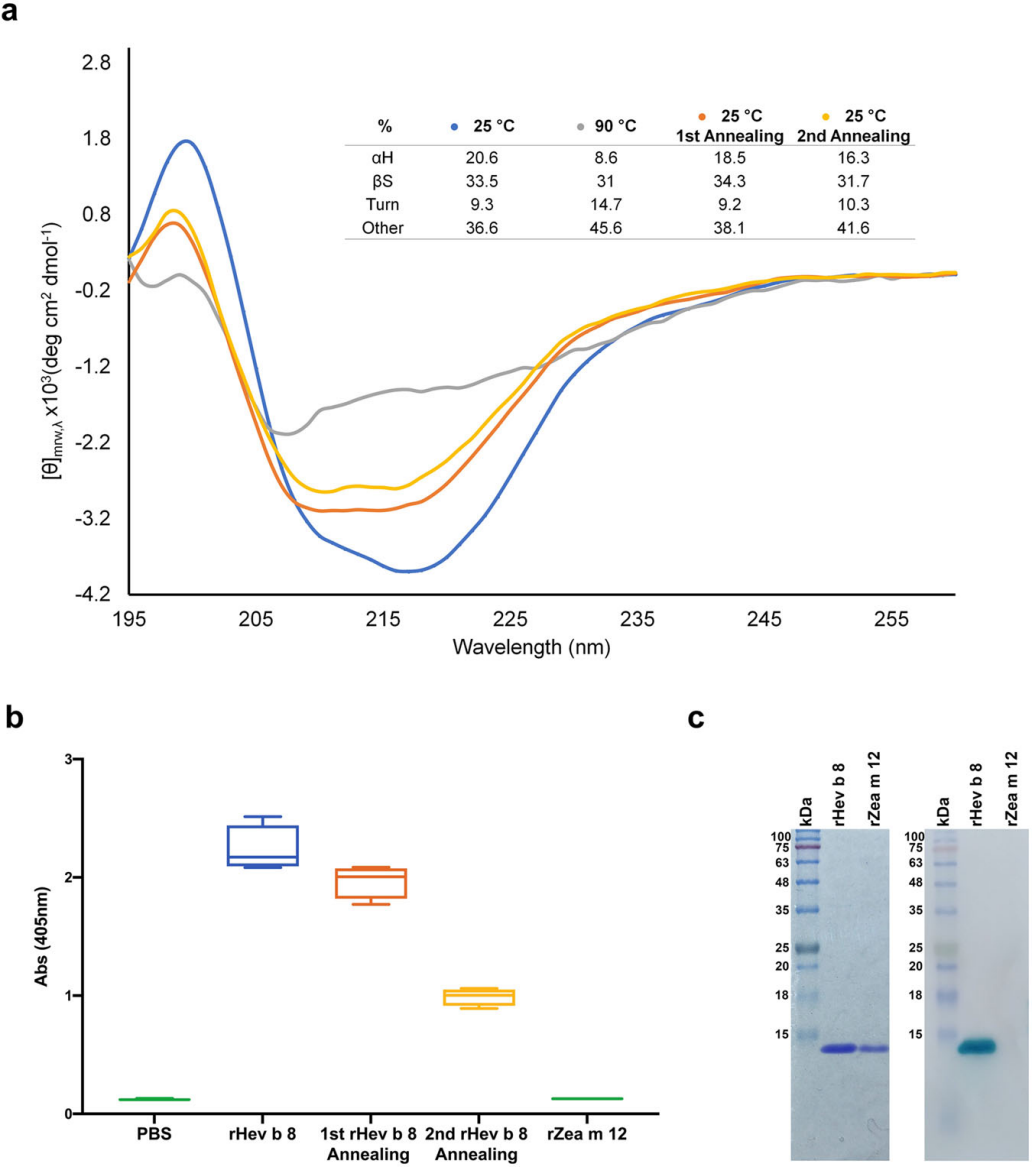
rHev b 8 profilin stability. **a** Far-UV CD spectra at different temperatures: at 25 °C before heat treatment (blue), at 90 °C (gray), and first (orange) and second (yellow) annealing processes (*n* = 3). The Table shows percentages of secondary structure (αH, α-helix; βS, β-sheet, Turns, and Other). **b** Box and whisker plot showing ELISA results of the recognition of rHev b 8 after the first and second annealing procedures, rZea m 12 was used as a negative control. Error bars represent standard deviations of the means of four replications. **c** Left: SDS-Page showing rHev b 8 and rZea m 12, Right: Western blot. IgE 2F5 recognized rHev b 8 but did not recognize rZea m 12.

The circular dichroism (CD) spectrum of rHev b 8 in the Far-UV region at 25 °C shows two negative bands centered at 218 and 208 nm and a positive band at 199 nm, indicating a significant amount of beta structures and alpha-helices. This result agrees with its 3D structure (PDB 5FDS). After two annealing procedures of increasing the temperature from 25 to 90 °C and cooling down slowly to 25 °C, the protein recovered most of its secondary structure. The band centered at 218 nm was modified after the thermal procedures; however, the alpha-helix content was recovered, although not entirely (Fig. 1a). Remarkably, through ELISA, we confirmed that rHev b 8 could be recognized by IgE 2F5, even after being subjected to the described annealing procedure twice. For ELISA, we used maize profilin (rZea m 12), which IgE 2F5 does not recognize, as a negative control (Fig. 1b)^20^. We confirmed through a Western blot experiment (Fig. 1c) that IgE 2F5 also recognizes rHev b 8 after treatment with denaturing agents and high temperature (SDS, and beta-mercaptoethanol, 95 °C).

### The structure of the Fab/IgE 2F5-rHev b 8 complex allows identification of the paratope-epitope pair and shows the basis of the allergen recognition

**Fig. 2:**
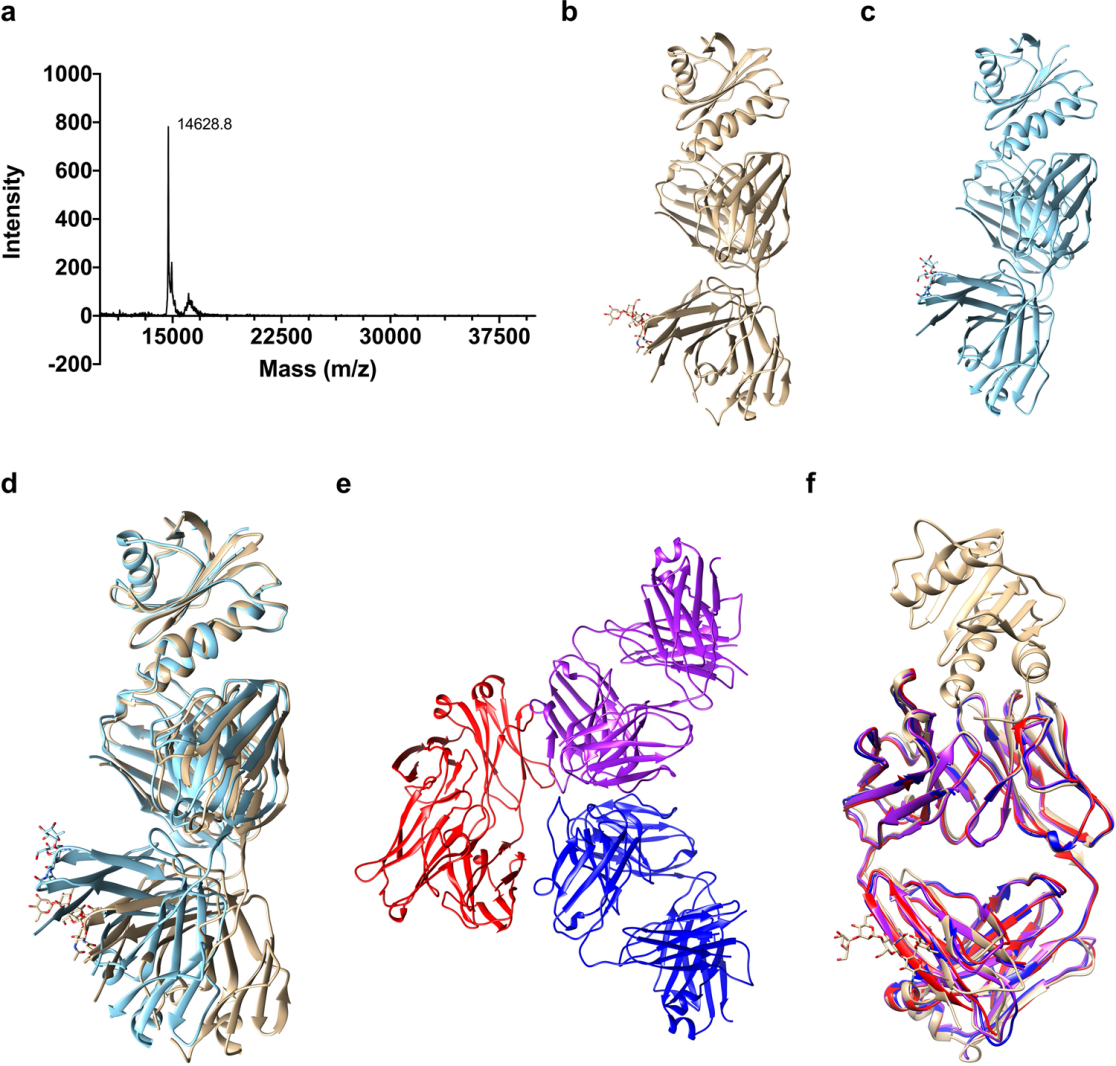
Basis of allergen recognition through the free Fab/IgE 2F5 and the complex with DDDK-rHev b 8 structures. **a** MALDI-TOF spectrum for the DDDK-Hev b 8 construct. **b** and **c** Ribbon diagrams of the complex Fab/IgE 2F5-DDDK-rHev b 8 at 3.04 (gold) and 3.34 Å resolution (blue), respectively. **d** Fab constant domains show a displacement of 19.2 degrees due to flexibility at the elbow region. **e** The three free Fab/IgE 2F5 molecules in the asymmetric unit (3.75 Å resolution). **f** Superposition of the three free Fab/IgE 2F5 molecules in the asymmetric unit (red, blue, and purple), with the complex Fab/IgE 2F5-DDDK-rHev b 8 at 3.04 Å resolution (gold).

To promote crystallization of the Fab/IgE 2F5-rHev b 8 complex, we used two different constructs, mature rHev b 8 and rHev b 8, with a short peptide (DDDK-rHev b 8) with a mass of 14,629 Da (Fig. 2a)^22^, as explained in the methodology. The difference of 474 Da corresponds to the last four residues of the enterokinase cleavage site (residues D-3, D-2, D-1, K-0). Therefore, we named this construct as DDDK-rHev b 8. Better diffracting crystals of the Fab/IgE 2F5-profilin complex were obtained with the DDDK-rHev b 8 construct. To understand the strong interaction between Fab/IgE 2F5 and this profilin, we determined two X-ray crystal structures of the complex, at 3.04 and 3.34 Å resolution, from crystals that grew in different conditions (Fig. 2b, c). The structures were obtained using the molecular replacement method. Both complexes crystallized in the orthorhombic space group P212121 with one copy of the Fab/IgE 2F5-profilin complex in the asymmetric unit. (Table 1 summarizes the data collection and refinement statistics).

**Table 1:** Data collection and refinement statistics (molecular replacement).

	Complex (PDB 7SBD)	Complex (PDB 7SBG)	FabIgE (PDB 7SD2)
Data collection			
Space group	P 21 21 21	P 21 21 21	I 1 2 1
Cell dimensions			
* a*, *b*, *c* (Å)	57.96, 77.47, 144.55	52.33, 71.45, 132.52	91, 103.9, 180.2
*α*, *β*, *γ* (°)	90, 90, 90,	90, 90, 90	90, 94.26, 90
Resolution (Å)	29.42–3.04	37.57–3.34	44.96–3.75
	(3.15–3.04)^a^	(3.46–3.34)	(3.88–3.75)
* R*_merge_	0.057 (0.27)	0.074 (0.438)	0.11 (0.44)
* I*/σ*I*	7.12 (1.93)	17.06 (2.89)	8.1 (2.3)
Completeness (%)	99.02 (93.21)	98.41 (95.33)	99.20 (99.20)
Redundancy	2.0 (2.0)	1.8 (1.8)	3.3 (3.3)
Refinement			
Resolution (Å)	29.42–3.04	37.57–3.34	44.96–3.75
	(3.15–3.04)	(3.46–3.34)	(3.88–3.75)
No. reflections	12929 (1181)	7616 (723)	17152 (1680)
* R*_work_/*R*_free_	22.77/25.38	24.54/29.59	24.66/28.43
No. atoms			
Protein	4017	3721	8148
Carbohydrate	60	38	0
Water	0	0	0
* B*-factors (Å^2^)			
Protein	63.74	72.91	102.02
Ligand/ion	80.26	91.64	
Water			
R.m.s. deviations			
Bond lengths (Å)	0.003	0.003	0.002
Bond angles (°)	0.65	0.64	0.57

^a^Values in parentheses are for the highest-resolution shell.

Both models showed the typical immunoglobulin fold of the constant and variable domains, and all complementarity-determining regions (CDR) of the heavy (VH) and light (VL) chains participate in the interaction with the allergen (Fig. 2b, c). A structural alignment of the models (Fab/IgE 2F5-rHev b 8) demonstrated flexibility at the Fab’s elbow region (Fig. 2d), corresponding to different conformational states in the crystals; nonetheless, the paratope region did not change, as has been reported by others^23^. In general, experimentally observed elbow angles for IgG Fabs vary by more than 15°^24^. The elbow angle for the structure at 3.04 Å is 131°, while the one at 3.34 Å is 113°. This difference was 19.2° in our models that crystallized under different conditions; this flexibility at the elbow region has not been reported for a Fab/IgE. We also found an N-linked glycosylation site in N189 of the Fab’s heavy chain constant region (Fig. 2b, c).

### The structure of Fab/IgE 2F5 indicates non-induced-fit allergen recognition

The overall structure of the free Fab/IgE 2F5 at 3.75 Å shows three Fabs molecules per asymmetric unit (Fig. 2e). We could not model all the residues of one of the three molecules in the asymmetric unit, but the electron density was enough to model all CDRs and the backbone. We compared the structure of the three free Fabs in the asymmetric unit with the Fabs structure in the rHev b 8 Fab/IgE 2F5 complex at 3.04 Å (Fig. 2f). No changes were observed, showing no flexibility, or induced fit in the antibody paratope when the allergen epitope was recognized. The free Fab/IgE 2F5 elbow region is comparable to the complex’s structure at 3.04 Å (RMSD varies from 0.73 Å for heavy chains to 1.1 Å for light chains) (Fig. 2f). Table 1 shows data collection and refinement statistics for these models.

### Fab/IgE 2F5 is glycosylated

**Fig. 3:**
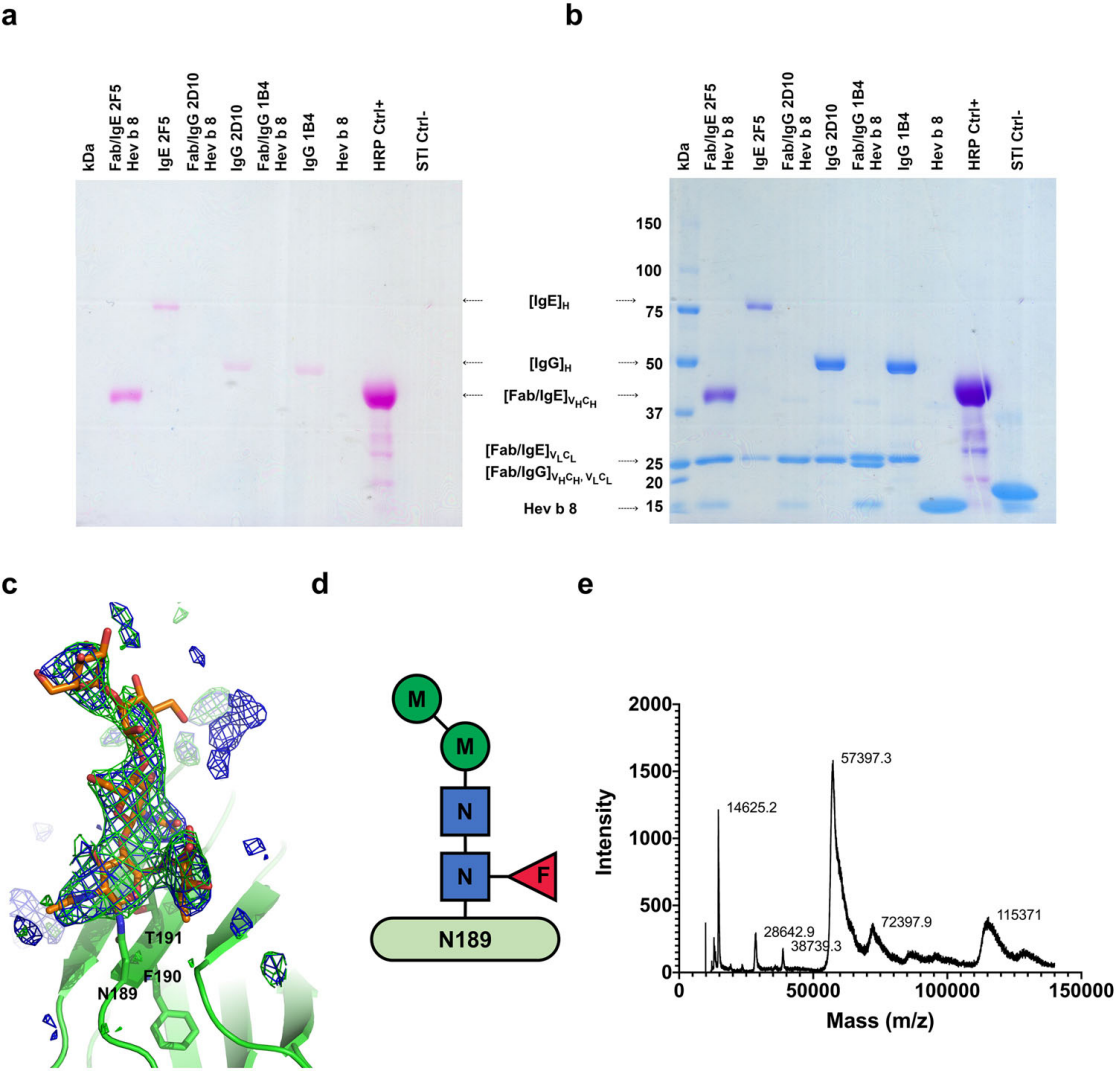
IgE 2F5 and its Fab are glycosylated. **a** SDS-PAGE stained with the Pierce Glycoprotein Staining Kit. Lane one: Molecular weight markers. Second lane: the Fab/IgE 2F5 heavy chain (VH and Cε1) is stained; Third lane: the complete IgE 2F5 heavy chain is stained; Fifth and Seventh lanes: Heavy chains of the complete IgG 2D10 and 1B4, which are glycosylated are stained. No stained bands were observed in the Fourth and Sixth lanes. Eighth and Tenth lanes, profilin (rHev b 8) and the soy trypsin inhibitor (STI) were used as negative controls: The Ninth lane shows horseradish peroxidase (HRP) used as a positive control. **b** The same gel was stained with Coomassie Blue G250. The Fab/IgE 2F5 light chain, IgG light and heavy chains, rHev b 8 profilin, and STI are observed. **c** Polder map contoured at 3σ (blue) and omit map 3σ (green) for the glycosylation at N189. **d** Composition of the oligosaccharide bound to N189 on the Fab/IgE 2F5, which comprises a core of di-mannose and fucose connected to the chitobiose core (GlcNAc2). Abbreviations: N, GlcNAc; M, mannose; F, fucose. **e** Mass spectra of DDDK-rHev b 8 (14,625 Da) and of the Fab/IgE 2F5 (57,397.3 Da). Lysozyme (14,100 Da) and bovine serum albumin (66,410 Da) were used to calibrate the spectrum. Interestingly, peaks of the Fab/IgE 2F5-DDDK-rHev b 8 complex (72,397.9 Da) and the dimeric Fab (115,371 Da) were also detected; however, these two peaks were not calibrated.

SDS-PAGE staining using the Pierce Glycoprotein Staining Kit showed that complete IgE 2F5 and its Fab were glycosylated (Fig. 3a). We detected glycosylation on the IgE 2F5 and its Fab heavy chains. In addition to horseradish peroxidase (44 kDa) and soy trypsin inhibitor (20.1 kDa), we used Fabs and complete murine IgGs 1B4 and 2D10^25^ as controls. Then, the same gel was stained using Coomassie Blue G250, and as shown in Fig. 3b, the blue staining of the other immunoglobulin fragments and the negative controls was observed. Murine IgG antibodies 1B4 and 2D10 were purified using the same procedure as IgE 2F5^25^. The crystal structure of Fab/IgE 2F5 showed N-linked main glycosylation at the N189/F190/T191 sequon of the Fab’s heavy chain constant region (Fig. 3c). Positive Fo-Fc electron density defined the branched sugar molecule bound at N189, which we interpreted as a core of N-linked N-acetylglucosamine (NAG) (β-1–4-linked), an α-1–6-linked fucose (FUC) bound to the first NAG, an a di-D-mannose (MAN) attached to the chitobiose core (GlcNAc2) (Fig. 3d), using the model-building tools implemented in Coot^26^. The Fab molecular mass determined by matrix-assisted laser desorption ionization-time-of-flight mass spectrometry (MALDI-TOF MS) was 57.4 kDa, while the value calculated from the sequence was 46.8 kDa (Fig. 3e). This result suggests that the difference of 10.6 kDa is probably due to posttranslational modifications. As three N-glycosylation sites have been reported for Fabs/IgE with oligosaccharide chains of approximately 2.2 kDa^27^, the modification’s difference in mass could be due to another type of posttranslational modifications present in murine antibodies or longer oligosaccharides conforming to the glycosylation chains. Interestingly, the mass measured for the whole rHev b 8 construct was lower than expected, with 14,625 Da instead of 18,283 Da. As reported previously, the rHev b 8 mass is 14,151 Da^20^. This difference corresponds to a construct with four additional residues at the N-terminal region (DDDK).

### Structural insights into profilin cross-reactivity

The DDDK-rHev b 8 model in the complex Fab/IgE 2F5-rHev b 8 exhibits three α-helixes and a β-sheet of seven β antiparallel strands connected by loops of different lengths, as has been reported previously for rHev b 8^20^. Profilin has a conformational epitope with a buried surface area of 926 Å^2^, as we determined using PDBsum^28^. This epitope involves conserved residues at the N- and C-terminal regions that correspond to the polyproline binding site in profilins^6^, and one residue in a loop that connects to β-strands 6 and 7.

**Fig. 4:**
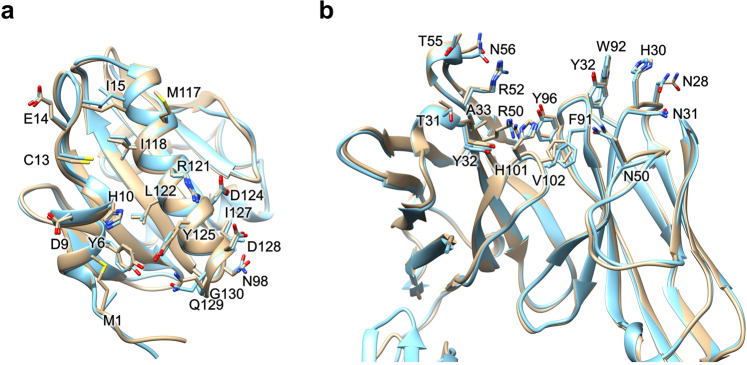
Paratope-epitope interactions. **a** Epitope residues. Structural alignment of the Fab/IgE-rHev b 8 models at 3.04 Å (gold) and 3.34 Å (blue). **b** Paratope residues alignment of the models at 3.04 Å (gold) and 3.34 Å (blue).

The rHev b 8 conformational epitope comprises 18 polar, acid, and basic residues. As shown in Fig. 4a, this epitope involves residues Y6, D9, H10, C13, E14, I15, M117, I118, L122, I127, Q129, and G130, which establish hydrophobic interactions and numerous nonbonded interactions (van der Waals and electrostatic) with the CDRs. In contrast, M1, N98, R121, D124, D128, and G130 form hydrogen bonds with residues in the CDRs. Meanwhile, D124 and D128 establish salt bridges with CDR-H2 residues R50 and R52, and the profilin Y125 establishes an aromatic interaction with Y32 (CDR-L1) (Supplementary Data 1). A hydrogen bond of 3.4 Å is formed between residues D-2 in rHev b 8 and the OH of Y49 in framework-L2.

The IgE 2F5 paratope involves the heavy chain residues T31, Y32, and A33 (CDR-H1); R50, R52, T55, and N56 (CDR-H2); and H101 and V102 (CDR-H3); and the light chain residues N28, H30, N31, and Y32 (CDR-L1); N50 (CDR-L2); and F91, W92, and Y96 (CDR-L3) (Fig. 4b).

The Fab/IgE 2F5 CDRs’ structural alignment showed that they all exhibit a very similar conformation in both structures (3.04 and 3.34 Å) (Fig. 4b). The interface interaction network in both structures is similar. Notably, this epitope region sequence is not entirely conserved in profilins from plants; however, it has been previously reported as a potential epitope for this allergen^29,30^. According to the sequence alignment of different plant profilins, several could cross-react with Hev b 8 (Supplementary Fig. 1).

**Fig. 5:**
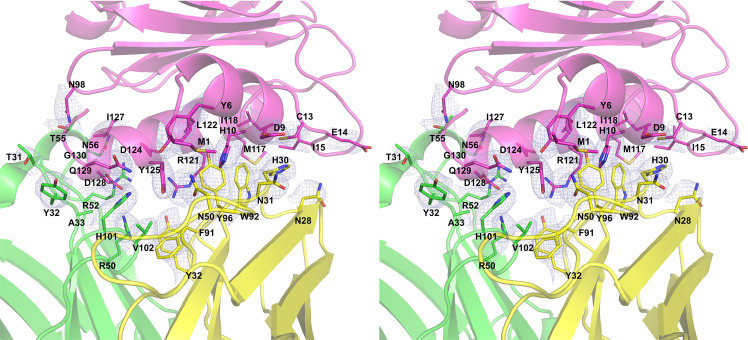
Stereo-view of the paratope-epitope interface. Fab/IgE 2F5 light chain (yellow), heavy chain (green) and profilin (pink), the 2Fo-Fc electron density map (1σ) is shown in light blue. The epitope-paratope interface area (926 Å^2^).

The Fab/IgE 2F5 paratope residues conform to a groove in which the hydrophobic and basic residues are surrounded by polar residues exposed to the solvent. Figure 5 shows the paratope-epitope surface displaying the groove formed mainly by the heavy chain. The epitope exhibits a complementary shape to the paratope and fits perfectly.

**Fig. 6:**
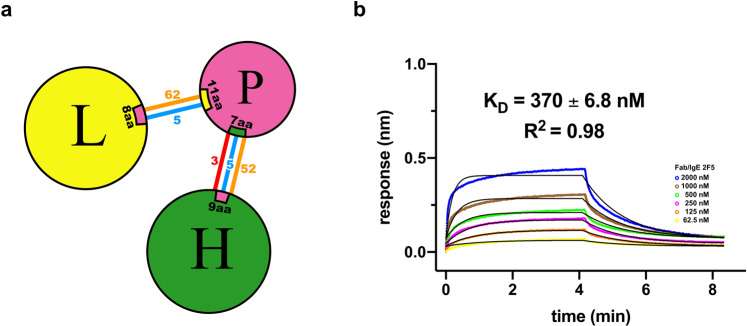
Paratope-epitope interactions and affinity of the Fab/IgE 2F5. **a** Interaction network summary. Heavy chain (H), light chain (L), and profilin (P). Salt bridges are represented as red lines, hydrogen bonds as blue lines, and nonbonded contacts (electrostatics and van der Waals interactions) with orange lines. **b** BLI fitting considering nonspecific binding (NSB) using the Association-Dissociation model implemented in GraphPad Prism 8 and a 1:1 model. Six different concentrations were tested and are shown in different colors.

According to the PIC (Protein Interaction Calculator)^31^ and PDBsum^28^ interaction analysis, summarized in Fig. 6a and detailed in Supplementary Data 1, the interactions established between rHev b 8 and the light and heavy chains of Fab/IgE 2F5 are three salt bridges, two cation-pi interactions, eight hydrophobic interactions, one aromatic interaction, numerous hydrogen bonds and nonbonded contacts (Electrostatic and van der Waals interactions).

### The affinity of the Fab/IgE 2F5 for rHev b 8

To compare the results of the interaction Fab/IgE 2F5-rHev b 8 with the previously reported results for IgE 2F5-rHev b 8, we used NaCl 1 M in the biolayer interferometry (BLI) experiments. In the absence of NaCl, no dissociation could be obtained; therefore, the salt concentration was increased up to 1 M to measure the dissociation and to prevent reassociation effects. The interaction between rHev b 8 and the Fab/IgE 2F5 analyzed using the (BLI) OCTECT software gave a dreadful adjustment with a 1:1 global fitting (Supplementary Fig. 2a). Therefore, we used the 1:1 global fit considering nonspecific binding (NSB) using the Association-Dissociation model implemented in GraphPad Prism 8^32,33^, obtaining a *K*_*D*_ value of 370 ± 6.8 nM (Fig. 6b). Even though it is not perfect, the fitting improved, as reported for Fab/IgG antigen interactions^34^. To confirm that the NSB fit does not affect the results, we also calculated the k_on_ (from the association step), then the *k*_off_ (from the dissociation step), separately, obtaining a *K*_*D*_ = 369 ± 3.8 nM (*k*_off_/*k*_on_) using Prism 8 (Supplementary Fig. 2b). Interestingly, the *K*_*D*_ value calculated using the IgE under the same conditions is approximately two orders of magnitude lower (1.7 nM)^20^.

### Generating cross-reactivity using profilin Zea m 12 that is not recognized by IgE 2F5

**Fig. 7:**
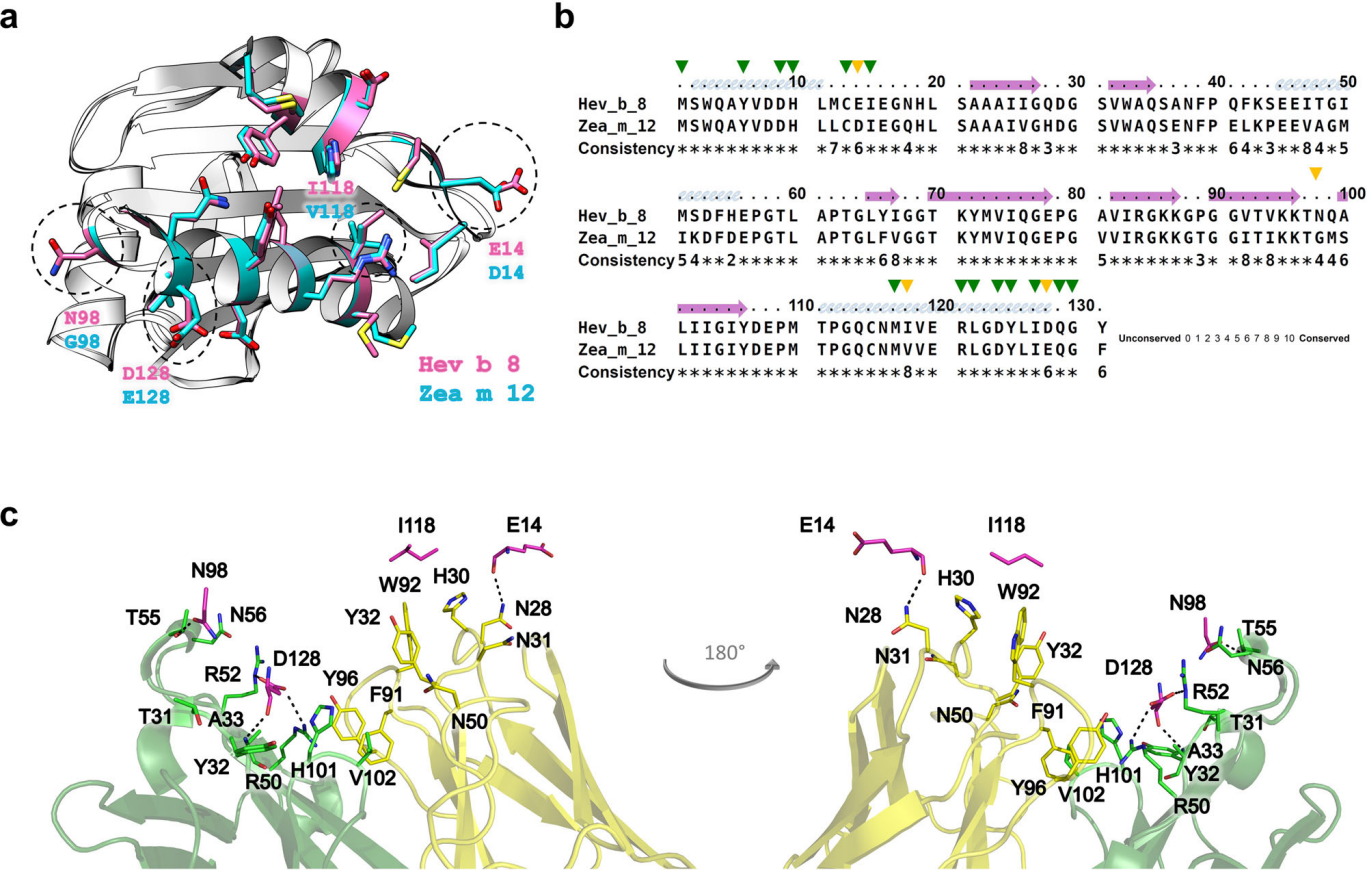
Generating cross-reactivity. **a** Ribbon and stick models of rHev b 8 (pink) and rZea m 12 (cyan) profilins (RMSD 0.307 Å). Different residues between both profilins (E14, N98, I118, and D128) are circled in dotted lines; this epitope region is part of the polyproline binding site in profilins. **b** Sequence alignment between rHev b 8 and rZea m 12, green arrows show conserved residues in the epitope, and yellow arrows show different residues. **c** Paratope residues of Fab/IgE 2F5 and the four residues in rHev b 8 that are different in rZea m 12 are displayed as sticks. rHev b 8 epitope residues are shown in pink, the Fab heavy chain residues in green, and the Fab light chain residues in yellow. Dotted lines indicate residues interactions.

We next attempted to produce and explain profilins’ cross-reactivity based on the obtained structural information. The sequence and structural alignments between rHev b 8 (PDB 5FDS) and rZea m 12 (PDB 5FEF) show the regions of high conservation involving the alpha-helices in the amino and carboxyl-terminal regions. We then identified four different residues in the epitope recognized by the Fab/IgE. rHev b 8 residues E14, N98, I118, and D128, which correspond to D14, G98, V118, and E128 in Zea m 12 (Fig. 7a, b).

Analysis of the structure of rHev b 8 bound to the Fab/IgE 2F5 supported that D128 on rHev b 8 is immersed in a complementary antibody cavity (Fig. 7c). D128 interacts with the paratope through R50 and R52 of the heavy chain establishing two salt bridges (Supplementary Data 1). Therefore, the presence of E128 in rZea m 12 exerts a significant steric hindrance. N98 (which corresponds with G98 on rZea m 12) is essential to stabilize the complex because it establishes a hydrogen bond and four nonbonded contacts with T55 of CDR-H2. Nonetheless, G98 does not establish any interaction with T55 (Supplementary Data 1), and its mutation is fundamental for recognition of Zea m12 by Fab/IgE 2F5. To test the relevance of residues D128 and G98 in the recognition of rHev b 8 by IgE 2F5 (and to understand the lack of recognition of rZea m 12 by the antibody), we first performed a single mutation E128D and then a double mutation E128D-G98N.

**Fig. 8:**
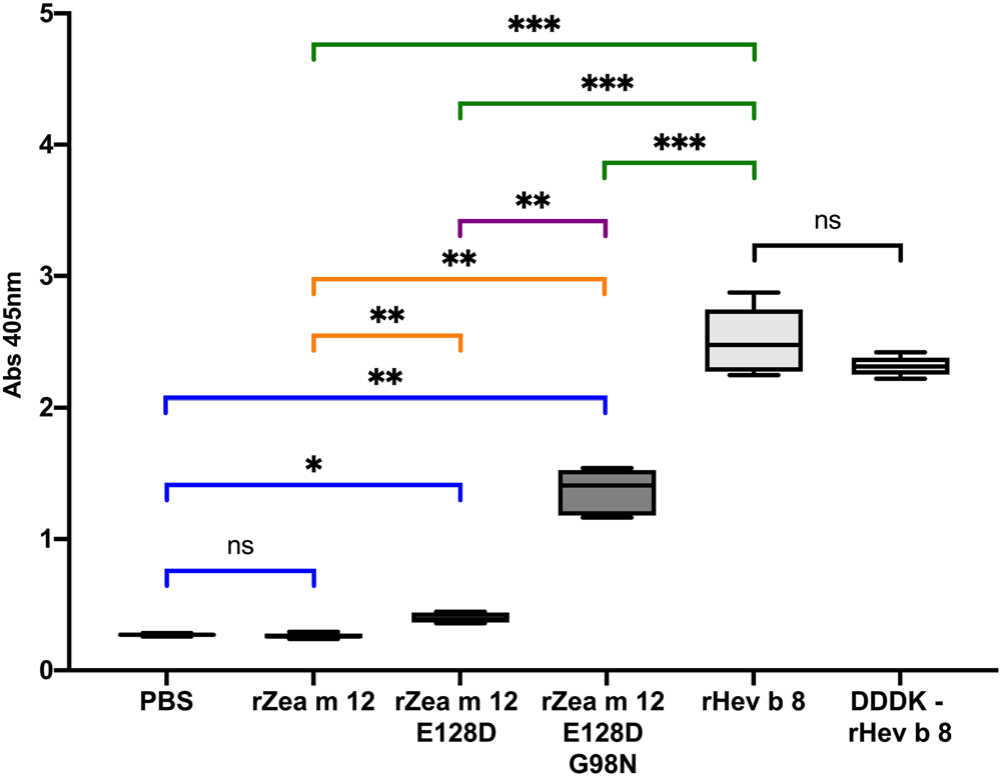
Cross-reactivity immunoassay. Box and whisker plot showing ELISA results for Zea m 12, and its mutants compared with rHev b 8 and the Sidak’s multiple comparisons tests. IgE interaction ELISA using rHev b 8, and the DDDK-rHev b 8 construct as positive controls, rZea m 12 as a negative control, rZea m 12-E128D single mutant, rZea m 12-E128D-G98N double mutant, and PBS as background control (ns not significant, all P values less than 0.001 are summarized with three asterisks). For these experiments, *n* = 4.

The immunoassay performed with the IgE 2F5 and the rZea m 12-E128D single mutant showed only a slight increase in recognition, barely above the background. However, the recognition of the rZea m 12 double mutant was significantly higher, reaching Abs values (405 nm) of approximately 50% of those observed for the binding of IgE 2F5 to rHev b 8 (Fig. 8). Regarding E14, no mutation was made because the interaction is established by the carbonyl group of the peptide bond. Likewise, the methyl group corresponding to the delta carbon of I118 does not cause a steric hindrance as E128 (Fig. 8).

## Discussion

The WHO/IUIS Allergen Nomenclature Sub‐Committee (http://www.allergen.org/) has reported natural and recombinant profilin Hev b 8 as an allergen able to trigger symptoms in latex-allergic patients. Profilins are ubiquitous proteins present in all eukaryotic cells; therefore, there is a high probability that individuals sensitized by one of the multiple isoforms of this protein will, in a subsequent exposure to other profilins, experience an allergic cross-reaction due to their high sequence identity, causing pollen-food syndrome^35^. In this study, we report the first crystal structures of the complex between a murine Fab/IgE (heavy and light chains naturally paired), bound to the allergen profilin from natural rubber latex (Hev b 8), and the structure of the Fab/IgE free.

The Fab/IgE 2F5-rHev b 8 complex structures revealed a conformational epitope constituted by the first and third α-helices of the allergen, corresponding to N- and C- terminal sequences that came close to each other in the three-dimensional structure (Fig. 4a), and a nearby residue in a loop connecting β strands 6 and 7. This region is rich in aromatic residues and constitutes the polyproline recognition site on profilins^36^, showing a conserved three-dimensional structure not necessarily reflecting identity in amino acid sequences. This epitope is unrelated to the loop comprising A37-S52, which we previously suggested as a possible epitope^20^.

A comparison of the two structures of the Fab/IgE 2F5-Hev b 8 complexes (at 3.04 and 3.34 Å resolution) shows that the Fab region of the murine IgE 2F5 exhibits flexibility at the elbow angle, comparable to that reported for Fabs from IgGs^23^; nonetheless, this flexibility does not affect the paratope (Fig. 2d).

Our Fab/IgE 2F5-rHev b 8 structure at 3.04 Å resolution revealed an electron density in both maps (2Fo-Fc and Fo-Fc) (Fig. 3c), interpreted as a branched oligosaccharide bound to N189. Reports demonstrate that murine and human IgEs exhibit three N-linked glycosylation sites in the VH domain^37^; however, its effect on antigen binding is unknown.

Some IgE characterizations performed by molecular modeling, FRET, and electron microscopy have also shown dynamic segmental flexibility and angle restrictions in the IgE conformation^38,39^. One of the most significant challenges in structure-function studies of IgEs is to have a simple way to observe different conformations at the atomic level to predict the rules that govern the intensity of an allergic response. A recent paper on the structure of an intact IgE and its interaction with the anti-IgE antibody ligelizumab, obtained by electron microscopy (EM) and small-angle X-ray scattering (SAXS), suggested a major IgE conformation with a defined Fab and Fc organization^10^. However, there are no structural studies of IgE-allergen complexes. Only two allergens complexed with chimeric IgG constructs containing human IgE variable regions have been reported^16,17^. Even though we do not have the structure of the intact IgE, our Fab/IgE structures provide valuable information concerning different conformations of this molecule, probably due to crystal packing, as suggested by others^23^, but causing minimal changes at the paratope.

It has been demonstrated that numerous allergens exhibit high stability under harsh conditions. The IgE of susceptible or allergic individuals can recognize allergens after a partial or total recovery of their three-dimensional structures^40,41^. According to our circular dichroism assays, obtained as a function of the temperature, rHev b 8 is an exceptionally stable panallergen. This profilin partially recovers its 3D structure but could presumably still trigger an allergic response after two annealing procedures since we detected 56% of the original IgE 2F5 recognition (Fig. 1a and b). Profilin stability is essential in antibody recognition and raises questions about why some profilins have been underestimated as relevant allergens. rHev b 8 is not the only profilin reported as thermostable; mustard profilin Sin a 4 was recognized by IgE from patients’ sera after thermal treatment^40^. This fact suggests that cooking food or subjecting allergens to other adverse conditions may not be enough to prevent an allergic response.

Functional Fabs from native, recombinant, or chimeric antibodies have been used in antibody structure characterization. The use of Fabs aims to decrease the intrinsic flexibility of whole antibodies. In our case, an intermediate interaction between Fab/IgE 2F5 and rHev b 8 was demonstrated using BLI experiments, considering that 1 M NaCl was present in the experiment. The Fab/IgE 2F5 *K*_*D*_ value of the interaction calculated using the 1:1 kinetic binding model was 370 nM (Fig. 6b). In contrast with the BLI experiment using the IgE^20^, fragmentation of this antibody affected the dissociation constant, most probably due to loss in avidity of bivalent IgE compared to the binding of the Fab/IgE. Compared with *K*_*D*_ value of the nonfragmented IgE 2F5 (1.7 nM), the Fab’s *K*_*D*_ is 200 times higher, indicating lower affinity.

As described above, complementary paratope-epitope surfaces explain the high affinity of IgE 2F5 for rHev b 8 (Fig. 5). When we performed the structural alignment of rZea m 12 over rHev b 8 bound to the Fab, we found a possible steric hindrance generated by E128 of rZea m 12, which corresponds to D128 in rHev b 8; that could help explain the absence of cross-reactivity (Fig. 7a). Furthermore, in rHev b 8, N98 (which corresponds with G98 on rZea m 12) is essential to stabilize the complex because it establishes a hydrogen bond and four nonbonded contacts with T55 of CDR-H2. The relevance of these residues for recognition of profilin by the 2F5 mAb was verified by creating two mutated rZea m 12: E128D and E128D-G98N. The results show that both residues (D128 and N98) are essential contributors to the high stability of the complex. However, this statement does not mean that other residues are not significant, as mutation of these two residues (E128 and G98) in rZea m 12 resulted in a significant recognition by IgE 2F5, albeit not to the same level as recognition of rHev b 8 (Fig. 8). According to Sidak’s multiple comparisons test^42^, IgE 2F5 recognition of the rZea m 12 mutants was significant compared to the recognition of rHev b 8, (Fig. 8). An extensive network of weak interactions at the interface paratope-epitope supports this result (Fig. 6a and Supplementary Data 1), where both profilins share 79.4% sequence identity.

Profilins have been underestimated as one of the most cross-reactive allergens and are currently classified as minor allergens based on their IgE-binding frequency. However, we demonstrated that annealing processes do not prevent the recognition of profilins by antibodies. Furthermore, we found using mutagenesis assays on the profilin from maize (rZea m 12), that minimal changes in aminoacidic sequence could avoid or allow cross-recognition of profilins by IgE antibodies.

The murine Fab/IgE 2F5 is a non-recombinant IgE obtained in response to immunization with rHev b 8 allergen. Therefore, VH and VL combination exhibits an authentic pairing of an IgE antibody. All CDR-H and CDR-L are essential for binding the surface of the profilin allergen. Further studies on the interaction of 2F5 (and other) anti-profilin antibodies with different profilins would help better define critical aspects of cross-reactivity among these significant allergens, which could have relevant implications in IgE engineering for diagnosis and research applications in allergy therapeutics.

## Materials and methods

### IgE 2F5 production and purification

We described the methodology for obtaining the IgE 2F5 in our previous paper^20^. Briefly, female Balb/c mice were subcutaneously immunized every two weeks for two months with the recombinant allergen rHev b 8 adsorbed in alum (Al (OH)_3_). We selected the mouse with a positive IgE response to the allergen for cell fusion. Ten days after the fusion, the supernatants of hybridoma cells were tested for the presence of IgE or IgG antibodies that recognized rHev b 8 using ELISA. We isolated one clone secreting IgE anti-profilin antibody (mAb 2F5), and the hybridoma secreting this IgE was cloned by limiting dilution. The isotype of the monoclonal IgE 2F5 was determined using the mouse immunoglobulin isotyping kit (Invitrogen, CA, USA).

Cell of the hybridoma 2F5 (producer of the monoclonal IgE antibody 2F5) were sent for sequencing of the Fv regions to Absolute Antibody Ltd (Redcar-Cleveland, United Kingdom), who provided the consensus sequences of the clone. Hybridoma cells^20^ grown in RPMI-1640 medium supplemented with sodium pyruvate, l-glutamine, nonessential amino acids, antibiotics, and 3% of fetal bovine serum (FBS) in a humid atmosphere at 37 °C with 5% CO_2_ produced the antibody. The secreted antibody was purified from the culture supernatant after centrifugation of the cell culture at 2000 × *g* for 20 min, and the cell pellet was discarded; the supernatant was filtered through a 0.22 μm membrane and applied to an affinity column equilibrated with PBS buffer, containing rHev b 8 covalently bound to an Affi-gel resin (Biorad, CA, USA). The antibody was eluted by changing pH with 0.2 M glycine-HCl buffer pH 2.8 and collected in 800 μL fractions in tubes containing 200 μL of 2.0 M Tris HCl buffer pH 8.0 to neutralize the pH immediately (Supplementary Fig. 3a).

### IgE fragmentation with papain

We performed the IgE fragmentation tests under the conditions established by Haba and Nisonoff, 1991^43^, (400 μL of papain, 75 μg/mL, 100 μL cysteine 0.5 M, 40 μL EDTA 0.5 M, 2 mg of IgE, 1 mL of PBS 10X, and 4.9 mL MilliQ water) incubated for 36 h at 37 °C. Murine IgG 2D10, previously obtained in our group^25^, was used as a positive control for papain digestion. The negative control was IgE incubated for 36 h without papain. Reactions were stopped by the addition of iodoacetamide to a final concentration of 20 mM. These samples were then applied to SDS-PAGE gels under reducing and nonreducing conditions, along with papain and both IgE and IgG without treatment as molecular mass controls.

We used papain from Sigma (P-4762, Sigma-Aldrich, St. Louis, MO, USA), and according to the supplier the enzyme cleavage site in our antibody is R204-T-I-L-V-R209 ↓ P210-V-N-I213, located between the Cε1 and Cε2 domains.

### Purification of the Fab/IgE 2F5 fragment

After papain hydrolysis, the IgE fragmentation mixture was applied to a Superdex 75 size exclusion column (SEC) in an AKTA-FPLC system at a 0.3 mL/min flow rate in 50 mM TRIS buffer, pH 8.4. The fractions displaying a protein band at 66 kDa in SDS-PAGE were concentrated in 10 000 MWCO diafiltration tubes (Sartorius, Göttingen, DE) and subsequently applied to a Mono Q GL cation exchange column, with a NaCl gradient from 0 to 250 mM in 40 mL. Finally, the fractions containing Fab were concentrated and dialyzed against crystallization buffer containing 20 mM TRIS-HCl and 50 mM NaCl pH 8.4, and purity was verified using SDS-PAGE (Supplementary Fig. 3b). The Fab/IgE 2F5 concentration was determined using the theoretical extinction coefficient at 280 nm calculated from the sequence (${A}_{280\,{{{{{{{\rm{nm}}}}}}}}}^{0.1 \% }$) 1.7 mL mg^−1^ cm^−1^.

### Protein expression and purification

#### rHev b 8 and rZea m12

Overexpression of rHev b 8 and rZea m 12 profilins was performed following the protocol described by Mares-Mejía, et al., 2016^20^ using the *Escherichia coli* Rosetta strain (DE3) transformed with the vectors pET-28c-rHevb8 or pET-28c-rZeam12. The transformed strains were inoculated in 50 mL of Luria Bertani liquid media (LB), supplemented with 50 μg/mL kanamycin and 34 μg/mL chloramphenicol, and incubated at 37 °C overnight. Subsequently, the culture was scaled to 1 L under the same conditions and induced with 0.5 mM β-D-1-thiogalactopyranoside (IPTG) at 30 °C for 12 h when the optical density was 0.7, at 600 nm. After the induction, the cells were harvested by centrifugation at 3100 × *g* for 10 min at 4 °C.

The cells were lysed in 50 mM TRIS-HCl buffer and 300 mM NaCl pH 8.0 and 1.0 mM phenylmethylsulfonyl fluoride (PMSF) with a Misonix 3000 sonicator (Misonix Inc. Farmingdale, NY, USA) using pulses of 10 s with power six and resting periods of 30 s until completing 10 min. The lysate was clarified by centrifuging at 3100 × g for 30 min, and the supernatant was collected. The supernatant was filtered through a 0.22 μm Millipore membrane and applied to a 5 mL HisTrap nickel affinity column (GE). Nonspecific interactions were eliminated by washing with 100 mL of 50 mM Tris HCl buffer, 300 mM NaCl, and 15 mM imidazole. Both recombinant proteins were eluted with 50 mM Tris HCl buffer, 300 mM NaCl, and 100 mM imidazole. Several milligrams of rHev b 8 whole construct was cleaved using EKMax^TM^, and all the rZea m 12 was cleaved using TEV protease (prepared in our lab). SDS-PAGE verified purity, and the rHev b 8 concentration was determined using (${A}_{280{{{{{{{\rm{nm}}}}}}}}}^{0.1 \% }$) of 1.1 mL mg^−1^ cm^−1^ for whole construct and 1.4 mL mg^−1^ cm^−1^ rHev b 8. The rZea m 12 concentration was determined using (${A}_{280{{{{{{{\rm{nm}}}}}}}}}^{0.1 \% }$) of 1.2 mL mg^−1^ cm^−1^ (Supplementary Fig. 3c, d).

#### Design and purification of the rZea m 12-E128D and E128D-G98N mutants

iPCR was performed using AccuPrime^TM^ Pfx Super mix (Invitrogen, Waltham, MA, USA) and the following primers (Sigma, St. Louis, MO, USA):

**E128D mutation**

SEQUENCE 5′-TAC CTG ATC GAT CAG GGC TTC-3′

COMPLEMENT5′-GAA GCC CTG ATC GAT CAG GTA-3′.

**E128D-G98N mutation**

SEQUENCE5′- C AAG AAA ACT AAC ATG TCC TTG-3′

COMPLEMENT5′- CAA GGA CAT GTT AGT TTT CTT G-3′

pET-28c-Zea m 12-E128D and pET-28c-Zea m 12-E128D-G98N plasmids were propagated in *E. coli* DH5α cells to be extracted, purified, and sequenced (Laragen, CA, USA). Purified vectors were transformed into *E. coli* Rosetta cells (DE3) to be overexpressed as previously described.

#### Effect of heat treatment on the rHev b 8 circular dichroism (CD) spectrum

The rHev b 8 CD spectra were obtained in the far-UV region (195–260 nm) using a JASCO J-1500 spectropolarimeter and a 1 mm path-length quartz cuvette. The protein concentration was 0.18 mg/mL in PBS, and the experiments were performed at 25 °C. The signal is expressed in terms of molar ellipticity θ (degree × cm^2^ × dmol^−1^). The baseline obtained using the buffer under identical conditions was subtracted from the final spectra. Three scans were averaged to obtain the final spectrum of the recombinant allergen.

We followed the thermal unfolding, setting the ellipticity at 218 nm while heating (25–90 °C) or cooling (90–25 °C) at one °C/min with a Jasco PTC-510 Peltier temperature controller and mini-Jasco MCB-100 water circulation bath. The spectrum was analyzed using the BeStSel online software^44,45^.

#### ELISA, Western blot assays and cross-reactivity

rHev b 8 thermal stability was evaluated by ELISA measuring the binding of IgE 2F5 to the different profilins. Plates of 96 wells were coated for two hours at 37 °C with 100 μL of 0.7 μM rHev b 8 or rZea m 12, in PBS buffer pH 7.4. Then, the wells were washed three times using PBS with 0.1% Tween 20 (washing buffer), blocked with 1% BSA in PBS, and incubated for two hours at 37 °C. The wells were washed three times, and 100 μL of IgE 2F5 diluted in PBS was added per well and incubated for one h at 37 °C. After incubation, the plate was washed three times with washing buffer, and 100 μL of HRP-labeled secondary antibody anti-mouse IgE Fcɛ specific (Abcam Inc., Cambridge, UK), diluted in PBS (1:3000), was added per well, and then incubated for one h at 37 °C. The peroxidase reaction was developed using 100 μL/well ABTS (Invitrogen, CA, USA) as the substrate and incubated for 20 min. Plates were read at 405 nm using a Cytation 3 plate reader (BioTek Instruments Inc. Winooski, VT, USA). The average absorbance of four independent value experiments and the corresponding standard deviation were plotted.

For western blot experiments, ten µg of rHev b 8 was electrophoresed on a 15% SDS-PAGE^25^. The gel was transferred to a PVDF membrane with a constant voltage (15 V) for 50 min. The PVDF membrane was blocked with 3% albumin, and 0.05% Tween 20 in PBS, washed three times using PBS with 0.1% Tween 20, and submerged in a two μg/μL IgE 2F5 solution overnight at 4 °C. The PVDF membrane was washed three times and then submerged in a secondary antibody anti-mouse IgE Fcɛ specific (Abcam Inc., Cambridge, UK) (1:2000 dilution) solution for 2 h at 37 °C. The PVDF membrane was washed three times, and the peroxidase reaction was developed using Novex HRP, and chromogenic substrate (TMB) (Pierce Thermo-Scientific, IL, USA) for 15 minutes.

To demonstrate cross-reactivity between rHev b 8 and rZea m 12, ELISA experiments were performed by coating the plate with 100 μL of 0.7 μM rHev b 8, DDDK-rHev b 8, rZea m 12, rZea m 12-E128D single mutant, or rZea m 12 -E128D-G98N double mutant, in PBS buffer pH 7.4. Phosphate-buffered saline (PBS) was used as a background control, DDDK-rHev b 8, and rHev b 8 were used as positive controls, and rZea m 12 was used as a negative control. We then followed the methodology previously described. The average of four independent determinations and the standard deviation were plotted.

#### Kinetic binding assays of Fab/IgE 2F5 with rHev b 8 using biolayer interferometry

Profilin rHev b 8 was biotinylated in PBS buffer pH 7.4, 0.05% Tween 20 and 1 M NaCl employing a 1:5 profilin: biotin ratio, and the excess of reactive ester groups were blocked using ethanolamine. Biotinylated profilin was dialyzed extensively to remove free biotin and ethanolamine using diafiltration tubes of 10,000 MWCO and immobilized to the streptavidin biosensor at a concentration of 125 nM. The assay was performed in a total volume of 200 μL using black bottom 96-well microplates (Merck KGaA, Darmstadt, Germany) at 25 °C with orbital shaking at 1000 rpm. The immobilized profilin was titrated with different concentrations of the Fab/IgE 2F5 (62.5, 125, 250, 500, 1000, and 2000 nM), BSA was used as the negative control, and new streptavidin biosensors were used for each experiment. All the experiments were performed using the Octet® RED96 System from FortéBio, controlled with the software Data Acquisition 8.2 (FortéBio Inc. San Jose, CA, USA). The BLI experiment baseline was PBS buffer pH 7.4, 0.05% Tween 20 and 1 M NaCl for 60 sec. The biotinylated allergen was then allowed to bind to the streptavidin sensor for 300 s, then washed with the same buffer to eliminate nonspecific binding. Next, the purified Fab/IgE 2F5 was bound to the allergen in the biosensor, and the association rate was measured (*k*_*a*_). The dissociation rate (*k*_*d*_) of the allergen-Fab complex was obtained in the last step. The data were processed using the Octet Data Analysis Software version 8.2 and fitted to 1:1. Besides, we used a BLI fitting considering nonspecific binding (NSB) through the Association-Dissociation model implemented in GraphPad Prism 8^33^ and a 1:1 model GraphPad Prism 8 association-then-dissociation model to fit the processed data.

#### Crystallization of the Fab/IgE 2F5-rHev b 8 complexes

The Fab/IgE 2F5-rHev b 8 complexes were prepared in two ways, using rHev b 8 and the DDDK-rHev b 8 (Supplementary Fig. 3d). The complex components were dialyzed in the crystallization buffer, mixed, and incubated for 16 h to form the complex. Profilin’s excess was removed using 50,000 MWCO diafiltration tubes and washed ten times with the crystallization buffer using a dilution factor of 10. The protein concentration was determined using (${A}_{280{{{{{{{\rm{nm}}}}}}}}}^{0.1 \% }$) of 1.66 mL mg^−1^ cm^−1^ and (${A}_{280{{{{{{{\rm{nm}}}}}}}}}^{0.1 \% }$) of 1.65 mL mg^−1^ cm^−1^ calculated for the complex using the rHev b 8 and DDDK-rHev b 8, respectively.

Both complexes’ crystallization screening was performed using a 1.7 mg/mL concentration with the Hampton Research PEG-Ion I and II crystallization kits and the sitting-drop vapor diffusion mode. After nine days, the complex formed by Fab/IgE 2F5 and the DDDK-rHev b 8 revealed crystals under various conditions. The best crystals were obtained with condition 24 of kit I (0.2 M lithium acetate, 20% PEG 3350). Using one of these crystals, we collected a dataset at 3.34 Å resolution employing a rotating anode generator MicroMax 007HF (Cu Kα, λ = 1.5418 Å), with a Dectris-Pilatus 3 R/200K-A detector. The crystal detector distance was 70 cm, with omega increments of 0.2° and an exposure time of 200 s per image. The cryoprotective solution was prepared with the stock solution and 30% glycerol.

After several months, we obtained an increasing number of crystals of the complex Fab/IgE 2F5 with the rHev b 8 DDDK-rHev b 8. We collected a dataset at 3.04 Å resolution from crystals that grew up in the condition containing 2% Tacsimate^TM^ pH 6.0, 0.1 M BIS-TRIS pH 6.5, 20% w/v Polyethylene glycol 3350. Data collection was performed using line 17-ID-1 of the National Synchrotron Light Source (NSLS) at the Brookhaven National Laboratory (BNL) in Upton, New York. We prepared the cryoprotective solution with the stock solution and 30% glycerol.

#### Crystallization of Fab/IgE 2F5

Fab/IgE 2F5 was dialyzed in 10 000 MWCO diafiltration tubes with ten washes of crystallization buffer II (20 mM TRIS-HCl with 200 mM NaCl, pH 8.4) using a dilution factor of 10. Subsequently, the Fab was concentrated to 3.3 mg/mL and screened using the PEG-Ion I and II crystallization kits (Hampton Research, Aliso Viejo, CA, USA) by vapor diffusion in the seated drop mode. Crystals appeared under various conditions after several months of incubation at 18 °C. The condition C1 (0.2 M magnesium acetate, 20% w/v polyethylene glycol 3,350) produced suitable crystals to be diffracted, and a dataset at 3.75 Å resolution was collected using a rotating anode generator MicroMax 007HF (Cu Kα, λ = 1.5418 Å) with a DECTRIS-PILATUS 3 R/200K-A detector. The crystal detector distance was 70 cm, with omega increments of 0.2° and an exposure time of 200 s per image. The cryoprotective solution was prepared with the stock solution and 30% glycerol.

#### Structure determination of the complex Fab/IgE 2F5-rHev b 8 and the Fab/IgE 2F5

The data collected at 3.04 and 3.34 Å were indexed, integrated, and scaled using the XDS^46,47^ and HKL3000^48^ software. Both crystals were orthorhombic and belonged to the space group (P212121). The structure of the complex Fab/IgE 2F5-DDDK-rHev b 8 at 3.04 Å was determined by molecular replacement with PHASER^49^ using rHev b 8 profilin (PDB 5FDS) and the anti-DNP Fab/IgE fragment (PBD: 1BAF) as search models. We performed iterative model building and refinement cycles with PHENIX Real-space refinement^50^, alternating manual model building with COOT^51^. The model was integrated onto the electron density maps 2Fo-Fc and Fo-Fc. Weight optimization of real-space refinement was performed during refinement. We used the coordinates of the complex Fab/IgE 2F5-rHev b 8 at 3.04 Å to determine the Fab/IgE 2F5-rHev b 8 structure using the crystal data at 3.34 Å resolution. For these models, the Ramachandran plots indicate 97.83% and 98.14% residues in favored region (2.17% and 1.86% in the allowed region) for the 3.04 and 3.34 models, respectively. Data processing and refinement statistics for both models are provided in Table 1. The Protein Interaction Calculator (PIC)^31^ and PDBsum^28^ were used to analyze the protein-protein interfaces.

The data collected at 3.75 Å for the Fab/IgE 2F5 were scaled using XDS^46,47^, and the space group was verified with Aimless^52^. The crystal was monoclinic and belonged to the space group (I121). The Matthews coefficient suggested three molecules in the asymmetric unit. The structure of Fab/IgE 2F5 was determined by molecular replacement with PHASER^49^ using the Fab structure of the complex Fab/IgE 2F5-rHev b 8 at 3.04 Å as the search model. We performed iterative refinement cycles using PHENIX Real-space refinement^50^, alternating manual model building with COOT^51^. The B-factors were refined as implemented in Phenix (XYZ coordinates, Real Space, Occupancies, and Individual B factor). In the final steps of the refinement, we set the value of all atoms to 50 Å^2^. Weight optimization of real-space refinement was also used during refinement. The model was integrated into the electron density maps 2Fo-Fc and Fo-Fc. For this model, the Ramachandran Plot indicates 97.03% residues in the favored region, whereas 2.97% in the allowed region. All the structure figures were generated using Chimera^53^ and PyMOL^54^. We used the Praline server^55^ for sequence alignments.

Atomic coordinates have been deposited in the Protein Data Bank under accession codes 7SBD (Complex at 3.04 resolution), 7SBG (Complex at 3.34 resolution), and 7SD2 (for the Fab).

#### Fab glycosylation detection by SDS-PAGE and the periodic acid-Schiff reagent

A gradient SDS-PAGE (7.5, 10 and 12% separation gel) was performed loading the Fab/IgE 2F5-rHev b 8 complex, IgE 2F5, Fab/IgG 2D10-rHev b 8 complex, IgG 2D10, Fab/IgG 1B4-rHev b 8 complex, IgG 1B4, profilin rHev b 8, horseradish peroxidase as a positive control and soybean trypsin inhibitor as a negative control. Glycoproteins were stained on gels with the periodic acid-Shiff reagent method was achieved using the Pierce Glycoprotein Staining Kit (Thermo Fisher, MA, USA).

#### Mass measurements by MALDI-TOF

We determined the molecular masses of recombinant rHev b 8 (whole construct), the Fab/IgE 2F5, and the complex Fab/IgE 2F5-rHev b 8 (whole construct) using matrix-assisted laser desorption/ionization-time of flight (MALDI-TOF) mass spectrometry (Microflex; Bruker Scientific LLC, Billerica, MA, USA). The standards for mass calibration were lysozyme (14,400 Da) and bovine serum albumin (BSA) (66,430 Da). The matrix used was a saturated solution of sinapinic acid in 30% (v/v) aqueous acetonitrile and 0.1% (v/v) trifluoroacetic acid. Samples were analyzed using FLEX ANALYSIS 3.0TM (Bruker) software.

#### Statistical analysis and reproducibility

The statistical analysis of the ELISAs was performed using the one-way analysis of variance (ANOVA), followed by an all-pairwise multiple comparison procedure (Šídák method)^42^. A *P* value < 0.05 was considered statistically significant. All summary statistics and analyses were performed using the Prism 8.0 software (GraphPad Software, La Jolla, Calif) (https://www.graphpad.com/). An asterisk identifies statistical significance and is denoted as *p < 0.05; **p < 0.01; ***p < 0.001; ****p < 0.0001. Error bars represent standard deviation from the mean. Sample size and replicates for each experiment are listed in the figure legends.

### Reporting summary

Further information on research design is available in the Nature Research Reporting Summary linked to this article.

## Supplementary information

Supplementary Information

Description of Additional Supplementary Files

Supplementary Data 1

Reporting Summary

## Data Availability

Atomic models for the complexes Fab/IgE-Hev b 8 have been deposited at the PDB with accession codes 7SBD and 7SBG (10.2210/pdb7SBD/pdb and 10.2210/pdb7SBG/pdb). The crystal structure of the Fab/IgE 2F5 has the PDB accession code 7SD2 (10.2210/pdb7SD2/pdb). Other data are available from the corresponding author on request.
