# Supplementary material for: A native IgE in complex with profilin provides insights into allergen recognition and cross-reactivity
Source: Commun Biol. 2022 Jul 27;5:748. doi: 10.1038/s42003-022-03718-w (PMC9334453; doi:10.1038/s42003-022-03718-w)
Supplement: Supplementary file 1 — Supplementary Information [file 42003_2022_3718_MOESM1_ESM.pdf]

Supplementary Information

A native IgE in complex with profilin provides insights into allergen recognition and cross-reactivity

Benjamín García-Ramírez[a], Israel Mares-Mejía[a], Annia Rodríguez-Hernández[a], Patricia Cano-Sánchez[a], Alfredo Torres-Larios[b†], Enrique Ortega[c], Adela Rodríguez-Romero[a*]

[a]Instituto de Química, Universidad Nacional Autónoma de México, Circuito Exterior, Cd. Universitaria, Coyoacán, Ciudad de México, 04510, México. [b]Instituto de Fisiología Celular, Universidad Nacional Autónoma de México, Circuito Exterior, Cd. Universitaria, Coyoacán, Ciudad de México, 04510, México. [c]Instituto de Investigaciones Biomédicas, Universidad Nacional Autónoma de México, Circuito Exterior, Cd. Universitaria, Coyoacán, Ciudad de México, 04510, México.

Corresponding author. Adela Rodríguez-Romero, PhD, Instituto de Química, UNAM, Circuito exterior, CU. Coyoacán 04510, CDMX, México. Email: adela@unam.mx

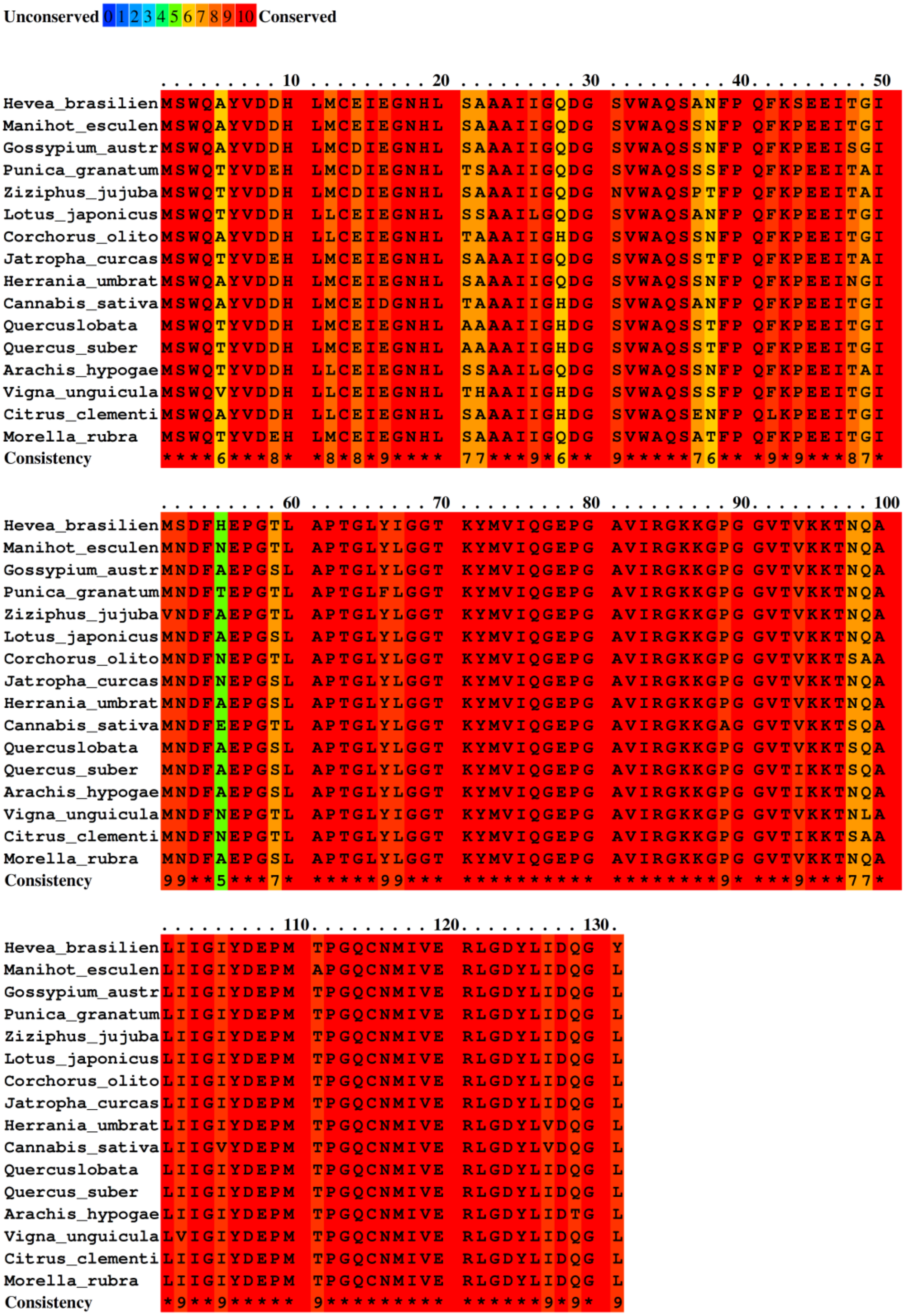

Supplementary Fig. 1. Sequence alignment of profilins that could cross-react with Hev b 8. Sequence alignment 15 was performed using the PRALINE server[37].

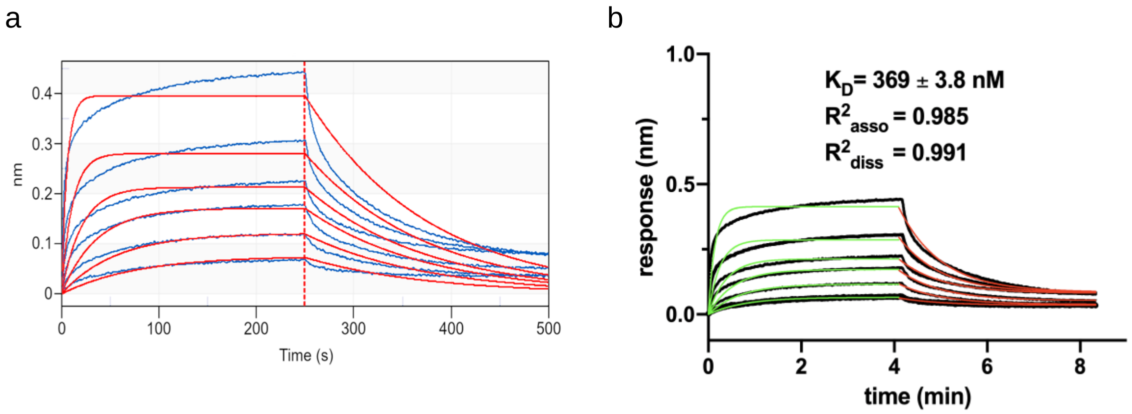

Supplementary Fig. 2. **BLI adjustments for the Fab/IgE 2F5 with rHevb 8**. a) BLI fitting using a 1:1 model as implemented in the BLI OCTECT software, with a 1:1 global fitting b) BLI fitting using GRAPHPAD PRISMA 8 considering NSB=0.

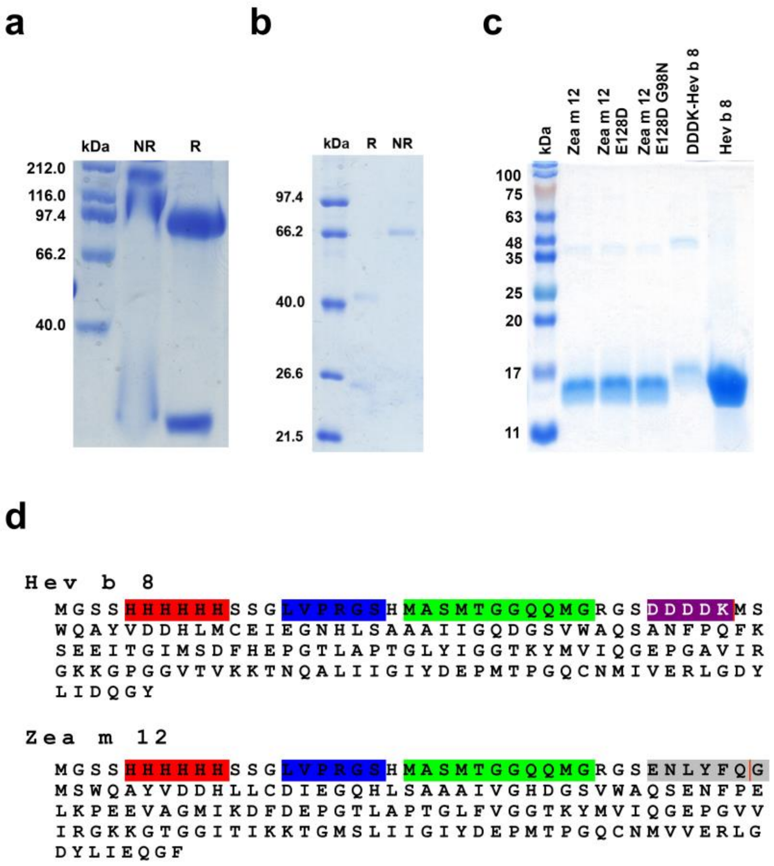

Supplementary Fig. 3. **SDS-PAGE under reducing conditions shows the heavy and light chains. a)** Purified IgE 2F5; **b)** Purified Fab/IgE 2F5 under reducing conditions (R) and non-reducing conditions (NR). **c)** Purified profilins. **d)** rHev b 8 and rZea m 12 whole constructions that contain at the N-terminal region a Histidine tag (red), a Thrombin cleavage site (blue), a T7 tag (green), an enterokinase cleavage site (purple in Hev b 8), and a TEV site (gray in Zea m 12).
