# Supplementary material for: A native IgE in complex with profilin provides insights into allergen recognition and cross-reactivity
Source: Commun Biol. 2022 Jul 27;5:748. doi: 10.1038/s42003-022-03718-w (PMC9334453; doi:10.1038/s42003-022-03718-w)
Supplement: Supplementary file 2 — Description of Additional Supplementary Files [file 42003_2022_3718_MOESM2_ESM.pdf]

Description of Additional Supplementary Files

File name: Supplementary Data 1
Description: Excell Table of the Hev b 8 – Fab/IgE 2F5 interactions.
