# Supplementary material for: A native IgE in complex with profilin provides insights into allergen recognition and cross-reactivity
Source: Commun Biol. 2022 Jul 27;5:748. doi: 10.1038/s42003-022-03718-w (PMC9334453; doi:10.1038/s42003-022-03718-w)
Supplement: Supplementary file 3 — Supplementary Data 1 [file 42003_2022_3718_MOESM3_ESM.pdf]

Supplementary Data 1. rHev b 8 – Fab/IgE 2F5 interactions

Hydrophobic Interactions within 5 Angstroms				
Fab/IgE 2F5			rHev b 8	
CDR-L1	32	Tyr	122	Leu
	32	Tyr	125	Tyr
FWR-L2	49	Tyr	1	Met
CDR-L3	91	Phe	125	Tyr
	92	Trp	117	Met
	92	Trp	118	Ile
	92	Trp	15	Ile
CDR-H3	102	Val	125	Tyr

π- π Interactions within 4.5 and 7 Å					
Fab/IgE 2F5			rHev b 8		Dist. [Å]
CDR-L1	32	Tyr	125	Tyr	5.3

Cation-Pi Interactions within 6 Å					
Fab/IgE 2F5			rHev b 8		Dist. [Å]
CDR-L1	32	Tyr	121	Arg	5.21
CDR-L3	96	Tyr	121	Arg	5.87

Salt Bridges							
Fab/IgE 2F5				rHev b 8			Dist. [Å]
CDR-H2	NH2	Arg	50	OD2	Asp	128	3.35
	NH2	Arg	52	OD1	Asp	124	2.88
	NE	Arg	52	OD1	Asp	128	2.98

Main Chain – Main Chain Hydrogen Bonds							
Fab/IgE 2F5				rHev b 8			Dist. [Å]
CDR-H1	33	Ala	N	128	Asp	O	3.28
	31	Thr	O	130	Gly	N	3.02

Main Chain - Side Chain Hydrogen Bonds							
Fab/IgE 2F5				rHev b 8			Dist. [Å]
CDR-H3	101	His	NE2	125	Tyr	O	3.44
CDR-L1	28	Asn	ND2	14	Glu	O	3.28
	28	Asn	ND2	14	Glu	O	3.28
	30	His	ND1	14	Glu	O	3.44
	32	Tyr	OH	122	Leu	N	3.36
FWR-L2	49	Tyr	OH	1	Met	N	3.45

CDR-L3	91	Phe	O	121	Arg	NE	2.99
	91	Phe	O	121	Arg	NH2	2.71
	91	Phe	O	121	Arg	NH2	2.71
	92	Trp	O	121	Arg	NE	2.99

Side Chain - Side Chain Hydrogen Bonds							
Fab/IgE 2F5				rHev b 8			Dist. [Å]
CDR-H2	50	Arg	NH2	128	Asp	OD2	3.35
	50	Arg	NH2	128	Asp	OD2	3.35
	52	Arg	NH2	124	Asp	OD1	2.88
	52	Arg	NH2	124	Asp	OD1	2.88
	52	Arg	NE	128	Asp	OD1	2.98
	52	Arg	NE	128	Asp	OD2	3.34
	55	Thr	OG1	98	Asn	OD1	2.77
	55	Thr	OG1	98	Asn	OD1	2.77
	55	Thr	OG1	98	Asn	OD1	2.77
CDR-H3	101	His	NE2	129	Gln	NE2	3.32
	101	His	NE2	129	Gln	NE2	3.32
	101	His	NE2	129	Gln	NE2	3.32
CDR-L2	30	His	ND1	13	Cys	SG	3.61
	30	His	ND1	13	Cys	SG	3.61
	32	Tyr	OH	10	His	ND1	3.45
	32	Tyr	OH	10	His	ND1	3.45
FWR-L2	49	Tyr	OH	-2	Asp	OD1	3.4
	49	Tyr	OH	-2	Asp	OD2	2.85
CDR-L2	50	Asn	ND2	1	Met	SD	3.09
	50	Asn	ND2	1	Met	SD	3.09
	50	Asn	OD1	125	Tyr	OH	3.22
	50	Asn	OD1	125	Tyr	OH	3.22
	50	Asn	OD1	125	Tyr	OH	3.22
CDR-L3	96	Tyr	OH	121	Arg	NH2	3.45
	96	Tyr	OH	121	Arg	NH2	3.45

Non bonded contacts							
Fab/IgE 2F5				rHev b 8			Dist. [Å]
	O	Thr	31	O	Ile	127	3.75
	O	Thr	31	C	Asp	128	3.74
	O	Thr	31	O	Asp	128	3.51
	O	Thr	31	C	Gln	129	3.33
	O	Thr	31	O	Gln	129	3.78
	O	Thr	31	N	Gly	130	3.02
	O	Thr	31	CA	Gly	130	3.45
	CA	Tyr	32	O	Asp	128	3.27

CDR-H1	C	Tyr	32	O	Asp	128	3.78
	CG	Tyr	32	O	Asp	128	3.87
	CD2	Tyr	32	O	Asp	128	3.46
	CE1	Tyr	32	O	Gln	129	3.67
	CE2	Tyr	32	CA	Gln	129	3.73
	CE2	Tyr	32	O	Gln	129	3.74
	CZ	Tyr	32	O	Gln	129	3.4
	OH	Tyr	32	O	Gln	129	3.59
	N	Ala	33	O	Asp	128	3.28
	CB	Ala	33	CB	Asp	128	3.79
	CB	Ala	33	CG	Asp	128	3.76
CDR-H2	NH2	Arg	50	OD2	Asp	128	3.35
	CD	Arg	52	OD1	Asp	128	3.7
	CD	Arg	52	OD2	Asp	128	3.53
	NE	Arg	52	CG	Asp	128	3.55
	NE	Arg	52	OD1	Asp	128	2.98
	NE	Arg	52	OD2	Asp	128	3.34
	CZ	Arg	52	OD1	Asp	128	3.83
	NH2	Arg	52	CB	Asp	124	3.51
	NH2	Arg	52	CG	Asp	124	3.27
	NH2	Arg	52	OD1	Asp	124	2.88
	NH2	Arg	52	OD1	Asp	128	3.88
	CB	Thr	55	OD1	Asn	98	3.53
	OG1	Thr	55	CG	Asn	98	3.83
	OG1	Thr	55	OD1	Asn	98	2.77
	CG2	Thr	55	OD1	Asn	98	3.4
	OD1	Asn	56	CG2	Ile	127	3.54
	OD1	Asn	56	CD1	Ile	127	3.84
CDR-H3	ND1	His	101	O	Asp	128	3.78
	ND1	His	101	CB	Asp	128	3.38
	CD2	His	101	CG	Gln	129	3.58
	CD2	His	101	NE2	Gln	129	3.59
	CE1	His	101	O	Tyr	125	3.2
	CE1	His	101	CD1	Tyr	125	3.9
	CE1	His	101	CB	Asp	128	3.44
	NE2	His	101	O	Tyr	125	3.44
	NE2	His	101	CD1	Tyr	125	3.69
	NE2	His	101	CE1	Tyr	125	3.42
	NE2	His	101	CZ	Tyr	125	3.62
	NE2	His	101	CG	Gln	129	3.42
	NE2	His	101	CD	Gln	129	3.88
	NE2	His	101	NE2	Gln	129	3.32
	CG1	Val	102	CE1	Tyr	125	3.42

	CG1	Val	102	OH	Tyr	125	3.67
CDR-L1	ND2	Asn	28	O	Glu	14	3.28
	O	His	30	O	Asp	9	3.71
	O	His	30	ND1	His	10	3.59
	O	His	30	CE1	His	10	3.85
	ND1	His	30	SG	Cys	13	3.61
	ND1	His	30	O	Glu	14	3.44
	CE1	His	30	O	Glu	14	3.13
	CE1	His	30	CA	Ile	15	3.79
	CE1	His	30	CB	Ile	15	3.62
	CE1	His	30	CG1	Ile	15	3.79
	NE2	His	30	CG1	Ile	15	3.74
	NE2	His	30	CD1	Ile	15	3.86
	C	Asn	31	CE1	His	10	3.88
	O	Asn	31	CE1	His	10	3.45
	O	Asn	31	NE2	His	10	3.83
	CB	Asn	31	O	Asp	9	3.84
	ND2	Asn	31	CB	Asp	9	3.85
	CD1	Tyr	32	ND1	His	10	3.81
	CD2	Tyr	32	ND1	His	10	3.74
	CE1	Tyr	32	ND1	His	10	3.36
	CE1	Tyr	32	CE1	His	10	3.85
	CE1	Tyr	32	CG	Tyr	125	3.82
	CE1	Tyr	32	CD1	Tyr	125	3.84
	CE2	Tyr	32	ND1	His	10	3.28
	CZ	Tyr	32	ND1	His	10	3.07
	CZ	Tyr	32	CG	Arg	121	3.85
	OH	Tyr	32	CB	His	10	3.75
	OH	Tyr	32	ND1	His	10	3.45
	OH	Tyr	32	CG	Arg	121	3.83
	OH	Tyr	32	N	Leu	122	3.36
	OH	Tyr	32	CA	Leu	122	3.33
	OH	Tyr	32	CB	Leu	122	3.45
	OH	Tyr	32	CD1	Leu	122	3.49
FWR-L2	CZ	Tyr	49	OD2	Asp	-2	3.84
	OH	Tyr	49	CG	Asp	-2	3.48
	OH	Tyr	49	OD1	Asp	-2	3.4
	OH	Tyr	49	OD2	Asp	-2	2.85
	OH	Tyr	49	CA	Lys	0	3.86
	OH	Tyr	49	C	Lys	0	3.66
	OH	Tyr	49	CB	Lys	0	3.2
	OH	Tyr	49	N	Met	1	3.45
	OH	Tyr	49	CB	Met	1	3.65

CDR-L2	CG	Asn	50	OH	Tyr	125	3.87
	OD1	Asn	50	CZ	Tyr	125	3.88
	OD1	Asn	50	OH	Tyr	125	3.22
	ND2	Asn	50	SD	Met	1	3.09
	ND2	Asn	50	CE	Met	1	3.8
	ND2	Asn	50	CE1	Tyr	6	3.81
	ND2	Asn	50	OH	Tyr	6	3.79
	ND2	Asn	50	OH	Tyr	125	3.86
CDR-L3	C	Phe	91	NH2	Arg	121	3.89
	O	Phe	91	NE	Arg	121	2.99
	O	Phe	91	CZ	Arg	121	3.28
	O	Phe	91	NH2	Arg	121	2.71
	C	Trp	92	NE	Arg	121	3.83
	O	Trp	92	CD	Arg	121	3.14
	O	Trp	92	NE	Arg	121	2.99
	CE3	Trp	92	CG	Arg	121	3.73
	CZ3	Trp	92	CB	Arg	121	3.79
	CZ3	Trp	92	CG	Arg	121	3.63
	OH	Tyr	96	CZ	Arg	121	3.87
	OH	Tyr	96	NH2	Arg	121	3.45
