# Supplementary material for: A native IgE in complex with profilin provides insights into allergen recognition and cross-reactivity
Source: Commun Biol. 2022 Jul 27;5:748. doi: 10.1038/s42003-022-03718-w (PMC9334453; doi:10.1038/s42003-022-03718-w)
Supplement: Supplementary file 4 — Reporting Summary [file 42003_2022_3718_MOESM4_ESM.pdf]

nature portfolio

Corresponding author(s): Prof. Adela Rodríguez-Romero

Last updated by author(s): Jun 24, 2022

Reporting Summary

Nature Portfolio wishes to improve the reproducibility of the work that we publish. This form provides structure for consistency and transparency in reporting. For further information on Nature Portfolio policies, see our Editorial Policies and the Editorial Policy Checklist.

Statistics

For all statistical analyses, confirm that the following items are present in the figure legend, table legend, main text, or Methods section.

n/a	Confirmed	
☐	☒	The exact sample size (*n*) for each experimental group/condition, given as a discrete number and unit of measurement
☐	☒	A statement on whether measurements were taken from distinct samples or whether the same sample was measured repeatedly
☐	☒	The statistical test(s) used AND whether they are one- or two-sided<br>*Only common tests should be described solely by name; describe more complex techniques in the Methods section.*
☒	☐	A description of all covariates tested
☐	☒	A description of any assumptions or corrections, such as tests of normality and adjustment for multiple comparisons
☐	☒	A full description of the statistical parameters including central tendency (e.g. means) or other basic estimates (e.g. regression coefficient) AND variation (e.g. standard deviation) or associated estimates of uncertainty (e.g. confidence intervals)
☐	☒	For null hypothesis testing, the test statistic (e.g. *F*, *t*, *r*) with confidence intervals, effect sizes, degrees of freedom and *P* value noted<br>*Give P values as exact values whenever suitable.*
☒	☐	For Bayesian analysis, information on the choice of priors and Markov chain Monte Carlo settings
☒	☐	For hierarchical and complex designs, identification of the appropriate level for tests and full reporting of outcomes
☒	☐	Estimates of effect sizes (e.g. Cohen's *d*, Pearson's *r*), indicating how they were calculated

Our web collection on statistics for biologists contains articles on many of the points above.

Software and code

Policy information about availability of computer code

Data collection	ELISA measured binding data were obtained using the BioTek Gen5 software. X-Ray Crystal diffraction data were collected using HKL-3000, BLI binding data were obtained using FortéBio Inc. Data Acquisition 8.2 Software. Profilin sequences for alignments were obtained using NCBI protein Blast
Data analysis	ELISA´s statistical analysis, using the measured binding data, was performed with Graphpad Prism 8. Crystal diffraction data were processed using XDS and HKL-3000. Phenix was used for molecular replacement and structure refinement. Manual model building was made using Coot. BLI binding data were fitted using GraphPad Prism 8. Sequence alignments were performed using the PRALINE server.

For manuscripts utilizing custom algorithms or software that are central to the research but not yet described in published literature, software must be made available to editors and reviewers. We strongly encourage code deposition in a community repository (e.g. GitHub). See the Nature Portfolio guidelines for submitting code & software for further information.

Data

Policy information about availability of data

All manuscripts must include a data availability statement. This statement should provide the following information, where applicable:

- Accession codes, unique identifiers, or web links for publicly available datasets
- A description of any restrictions on data availability
- For clinical datasets or third party data, please ensure that the statement adheres to our policy

https://doi.org/10.2210/pdb7SBD/pdb; https://doi.org/10.2210/pdb7SBG/pdb; https://doi.org/10.2210/pdb7SD2/pdb

Field-specific reporting

Please select the one below that is the best fit for your research. If you are not sure, read the appropriate sections before making your selection.

☒ Life sciences ☐ Behavioural & social sciences ☐ Ecological, evolutionary & environmental sciences

For a reference copy of the document with all sections, see nature.com/documents/nr-reporting-summary-flat.pdf

Life sciences study design

All studies must disclose on these points even when the disclosure is negative.

Sample size	Size of the samples were properly selected according with other similar reports across the world
Data exclusions	No data was excluded in the analysis
Replication	ELISA were performed using four independent determinations.
Randomization	Randomization was not necessary because all the experiments were performed in-vitro following a set of controlled techniques.
Blinding	N/A

Reporting for specific materials, systems and methods

We require information from authors about some types of materials, experimental systems and methods used in many studies. Here, indicate whether each material, system or method listed is relevant to your study. If you are not sure if a list item applies to your research, read the appropriate section before selecting a response.

Materials & experimental systems

n/a	Involved in the study	
☐	☒	Antibodies
☐	☒	Eukaryotic cell lines
☒	☐	Palaeontology and archaeology
☒	☐	Animals and other organisms
☒	☐	Human research participants
☒	☐	Clinical data
☒	☐	Dual use research of concern

Methods

n/a	Involved in the study	
☒	☐	ChIP-seq
☒	☐	Flow cytometry
☒	☐	MRI-based neuroimaging

Antibodies

Antibodies used	IgE 2F5, IgG 2D10, IgG 1B4, were produced in our lab. Mouse monoclonal [b3102e8] anti human IgE Fc (HRP) from Abcam
Validation	Absolut Antibody Ltd. provided the Fab region consensus sequence of the IgE 2F5, from murine hybridoma. GenScript provided the Fv region consensus sequences of the clones IgG 2D10 and IgG 1B4.<br>https://doi.org/10.1038/srep32552 - Structural insights into the IgE mediated responses induced by the allergens Hev b 8 and Zea m 12 in their dimeric forms<br>https://doi.org/10.1016/j.molimm.2020.09.017 - Novel murine mAbs define specific and cross-reactive epitopes on the latex profilin panallergen Hev b 8.

Eukaryotic cell lines

Policy information about cell lines

Cell line source(s)	Murine hybridomas.
Authentication	None of the hybridome cell lines used were authenticated.
Mycoplasma contamination	Cells lines were not tested for mycoplasma contamination
Commonly misidentified lines (See ICLAC register)	N/A
